# Functional diversities of myeloid cells in the central nervous system

**DOI:** 10.1111/cns.13525

**Published:** 2020-11-17

**Authors:** Wenting Zhang, Tarun N. Bhatia, Rehana K. Leak

**Affiliations:** ^1^ Pittsburgh Institute of Brain Disorders & Recovery and Department of Neurology University of Pittsburgh Pittsburgh PA USA; ^2^ Graduate School of Pharmaceutical Sciences Duquesne University Pittsburgh PA USA

## 1 MYELOID CELLS IN THE BRAIN

Microglia were first defined by Pío Del Río‐Hortega circa 1919, based on their small somas, phagocytic functions, and mesodermal origin. Distinct from other central nervous system (CNS)‐resident glia, microglia originate from embryonic myeloid progenitors in the yolk sac that migrate via the blood into the CNS before closure of the blood‐brain barrier (BBB). In the CNS, newly generated microglia continue their proliferation and spatial distribution during development. As with other immune cells, the self‐renewal ability of microglia is preserved throughout life, a function that may be exploited for reestablishing homeostatic equilibria after CNS injury or disease onset.

Apart from the abundant parenchymal microglia, non‐parenchymal, border‐associated myeloid cells (BAMs) are derived from erythromyeloid precursors and reside in the meninges, choroid plexus, and perivascular spaces. Although BBB closure blocks circulating monocytes from invading the CNS, macrophages in the choroid plexus and dura mater may be replaced by monocytes through adulthood. Further, BBB breakdown in disease facilitates infiltration of circulating myeloid cells, such as monocytes, granulocytes, and dendritic cells from the vasculature into the brain, where they initiate or partake in inflammatory responses.

Border‐associated myeloid cells share some prototypical genetic markers with microglia, such as *Sall1*, *Slc2a5*, *Olfml3*, and *Tmem119*, but segregate in expression of *Apoe*, *Ms4a7*, *Ms4a6c*, and *Clec4a*. Based on heterogeneities in their genetic signatures, one can infer functional diversities of CNS myeloid cells at all stages of development and aging.

## 2 FUNCTIONAL HETEROGENEITIES OF MICROGLIA AND THEIR MOLECULAR MEDIATORS

Recently, the spatiotemporal heterogeneity of microglia was investigated by single‐cell RNA‐seq.^1^ Lysosome‐related genes were highly expressed by embryonic microglia, which need to engage in phagocytosis of apoptotic neurons. In contrast, postnatal microglia express homeostatic genes, including *Tmem119*, *Selplg*, and *Slc2a5*. Further, microglia in white matter (WM) show higher levels of phagocytosis‐related markers than in gray matter, due perhaps to differential effects of the surrounding oligodendrocyte precursor cells within WM.^2^ Spatiotemporal diversities in microglial function are thus also likely to exert reciprocal effects on the specific milieu and cell types with which these glia interact.

Quiescent microglia (M0) with ramified morphologies display highly dynamic behaviors in vivo, frequently extending thin processes for surveillance, and are stabilized by receptors such as TREM2, CX3CR1, CSF‐1R, and CD200R. CX3CR1 deficiency transiently inhibits microglia, reducing synaptic pruning,^3^ whereas CD200 deficiency activates microglia by CD11b and CD45 upregulation.^4^ Activated microglia are rational targets for the treatment of stroke, traumatic brain injury (TBI), and neurodegenerative disorders. Indeed, anti‐inflammatory drugs may improve clinical outcomes and microglial inhibition may be neuroprotective in some disease models.^5^ Other reports show that *non‐specific* microglia depletion exacerbates neurological deficits.^6^ Thus, the role of microglia in the diseased brain remains a topic of contention and microglial functional heterogeneities need to be established in physiological versus pathological contexts. Classically activated, M1‐polarized microglia exacerbate neuronal death by pro‐inflammatory molecules, such as TNFα, IL‐1β, and free radicals, whereas alternatively activated M2 microglia contribute to repair by clearing debris, producing trophic factors, and pro‐resolving mediators. However, these states were defined based on stimulation with single cytokines in vitro. Microglia are likely to be more heterogenous in vivo, existing along a continuum of states rather than in discrete M0/M1/M2 phenotypes.

Our previous studies revealed that, in the acute stages after ischemic injury, microglia express M2‐phenotypic markers (CD206, Arg1), which are gradually replaced by pro‐inflammatory, M1‐phenotypic markers, such as CD16 and CD86.^7^ In contrast, intracerebral hemorrhage initially skews microglia toward M1, with M2 microglia accumulating in the subacute phase, contributing to clearance of the hematoma. BBB breakdown leads to infiltration of bone marrow–derived circulating myeloid cells into injured parenchyma, accelerating angiogenesis, WM regeneration, and debris clearance. However, pro‐inflammatory factors released by these myeloid cells may induce secondary injuries. Thus, the collateral damage introduced by myeloid cell invasion of the CNS warrants further investigation.

Toll‐like receptors play critical roles in microglial activation following ischemia. Stroke induces TLR4, which activates nuclear factor kappa‐light‐chain‐enhancer of activated B cells (NF‐κB). NF‐κB activation promotes M1 polarization, and peroxisome proliferator‐activated receptor γ (PPARγ) serves as a gatekeeper to M2 polarization by blocking the NF‐κB pathway.^8^ Thus, PPARγ agonists protect against acute brain injuries by suppressing pro‐inflammatory cytokines, including TNFα, IL‐β, IL‐6, and boosting anti‐inflammatory cytokines, such as IL‐4, IL‐10, TGF‐β, and IGF‐1.

In Alzheimer's disease, Aβ plaques activate microglia, promoting cytotoxicity, but a recent study identified a novel subset of protective, disease‐associated microglia (DAM) that emerge by downregulation of microglia‐specific inhibitory checkpoints.^9^ Single‐cell RNA‐seq shows that phagocytic genes are upregulated in DAM and this transition of homeostatic microglia to DAM may be dependent on the recognition of “danger signals” or neurodegeneration‐associated molecular patterns by surface receptors (eg, TREM2).^9^ Thus, microglia harbor the intrinsic machinery required to trigger their transformation into protective phenotypes, although the underlying mechanisms are less known, but may be novel targets for the development of therapies for CNS diseases.

Functional microglial diversity is influenced not only by disease, but also by age, biological sex, and other factors such as stress and diet. Microglial depletion and repopulation have emerged as a potential strategy to facilitate brain repair and reverse age‐induced neuronal and cognitive deficits. Sexual dimorphisms in microglia function in stroke have been demonstrated by favorable effects of transplantation of female microglia into the male brain.^10^ On the other hand, the contribution of these sex differences in microglial function to the onset and severity of various age‐related neurodegenerative diseases is unclear and warrants further study.

In sum, microglial heterogeneities in structure and function are heavily context‐dependent. To optimize the therapeutic efficacy of microglia, we will need to learn how to fine‐tune the precise molecular mechanisms that control their functional diversities.

## CONFLICT OF INTEREST

The authors declare no conflict of interest.
